# Drug-related proteinuria: a vigilance analysis based on the FAERS database

**DOI:** 10.1590/1414-431X2025e15097

**Published:** 2026-03-02

**Authors:** Zhixiang Mao, Linjian Zhou, Yi Nie, Hongbing Wang, Junying Zhang

**Affiliations:** 1 Affiliated Hospital of Xuzhou Medical University, Xuzhou, China; 2 Jiangsu Cancer Hospital, Nanjing, China

**Keywords:** Adverse events, Proteinuria, FAERS, Acute kidney injury, Cancer

## Abstract

Proteinuria is a prevalent and significant adverse response (ADR) associated with numerous pharmaceuticals, and we employed the online public FDA Adverse Event Reporting System (FAERS) database to investigate a cohort of medications that may induce this ADR. This analysis aimed to identify and assess the most prevalent and significant medicines linked to the risk of proteinuria. We examined the publicly accessible FAERS database from 2004 to 2024. Utilizing the search term “proteinuria” and classifying by generic drug name, we aggregated reports of drug-related responses or trends in proteinuria, subsequently analyzing the data through a combination of ratio-of-reported-ratio (ROR) and proportional-reported-ratio (PRR) to identify and examine twelve medications that may induce proteinuria. A total of 16,355 adverse event reports related to proteinuria were identified in the FAERS database between 2004 and 2024. Among these, 21 drugs demonstrated statistically significant associations with proteinuria based on multivariate logistic regression, with the highest signals observed for voclosporin (ROR: 63.57) and lenvatinib (ROR: 41.01). Drug classes most strongly associated included anticancer agents, immunosuppressants, and antiviral drugs. Notably, the onset of proteinuria varied significantly across drug types, with anti-inflammatory agents showing the earliest median onset (5.4 days), while digestive system drugs and antivirals exhibited delayed onset exceeding 1,000 days on average. These findings underscore the need for early and long-term renal monitoring depending on drug category. Prompt assessment of nephrotoxicity risk is essential during the initial phase of medication, hence offering a more precise foundation for drug screening and optimization.

## Introduction

Proteinuria is an important marker of renal injury, and its presence often implies that renal filtration and reabsorption are impaired. Prolonged proteinuria can be detrimental to the kidneys in several ways (1). Protein filtration from the glomerulus into the renal tubules has a toxic effect on renal tubular epithelial cells, triggering an inflammatory response that leads to tubular atrophy and interstitial fibrosis. This accelerates glomerulosclerosis, ultimately causing a gradual decline of renal function and the development of chronic renal failure (2,3). In addition, proteinuria also has a significant impact on the whole body; a large amount of protein is lost from the urine, which will lead to hypoproteinemia, causing malnutrition, and pediatric patients may experience developmental delays (4). Proteinuria can reduce the body's immunoglobulin levels, decrease immunity, increase susceptibility to a variety of pathogens, and make infections more difficult to treat. Insufficient protein in the body may also induce endocrine disorders, such as T3 syndrome. Hypoproteinemia is often accompanied by hyperlipidemia (5). Increased blood viscosity increases the risk of thromboembolic complications (6), posing a serious threat to the patient's life and health.

Drug-related proteinuria is a major cause of secondary proteinuria, characterized as a condition in which the use of drugs raises the protein concentration in urine above normal limits, commonly indicating kidney damage (7,8). About 14-26% of acute kidney injury cases are linked to adverse drug reactions, with proteinuria serving as an early marker of nephrotoxicity, often indicating potential damage to the glomerular or renal tubular interstitial structures (9). Drugs induce proteinuria through direct and indirect mechanisms. Nonsteroidal anti-inflammatory drugs directly damage renal tubular epithelial cells, resulting in leakage of low molecular weight proteins (10). Immune checkpoint inhibitors may increase glomerular filtration barrier permeability by altering glomerular hemodynamics or inducing immune complex deposition (11). Chemotherapy agents, such as cisplatin and bevacizumab, exacerbate podocyte damage via oxidative stress or inhibition of the vascular endothelial growth factor (VEGF) signaling pathway (12). The clinical presentation of drug-induced proteinuria is often insidious and delayed. If not identified and managed early, it can progress to irreversible chronic kidney disease.

Proteinuria-related adverse events have increased dramatically in recent years. Thus, clinicians should improve their awareness of drug nephrotoxicity in order to limit the occurrence of drug-related kidney impairment. Our team assessed the extent of reporting of various medicines related to proteinuria responses using publicly available FDA Adverse Event Reporting System (FAERS) data (13). Various drugs that may cause drug-related proteinuria are listed and discussed, and the possible causes of such adverse events are analyzed. The aim was to provide clinicians with more references to adverse events, emphasize the importance of careful medication use, strengthen patient education on medication use, closely monitor adverse events, and adjust the method of administration in a timely manner to ensure medication safety.

## Material and Methods

### Data source and study design

We systematically enumerated and downloaded all data from the FAERS database spanning from the first quarter of 2004 to the end of 2024. Each file comprised eight distinct data types, including DEMO, which contains patient demographic and administrative information for each reported event record; REAC, which includes all Medical Dictionary of Regulatory Activities (MedDRA) terms coded for an event; DRUG, which provides drug and biologic information for the most reported drugs associated with an event; and OUTC, which details the final outcomes for patients involved in an event. The DRUG table includes drug and biological information for most drugs reported in the event. The OUTC table presents the final outcomes for patients associated with the event. The RPSR table details the sources of event reports. The THER table records the start and end times of treatment for the reported drug. Lastly, the INDI table encompasses all MedDRA terms coded for the reported medication use and diagnostic indications. All data were imported into PostgreSQL. Duplicates were eliminated to maintain the accuracy and precision of the data. Adverse events (AEs) in REAC can be classified using the MedDRA preferred terminology (PT). The five primary categories of proteinuria are glomerular proteinuria, tubular proteinuria, mixed proteinuria, overt proteinuria, and tissue proteinuria. Events related to proteinuria were classified into four categories: primary suspect, secondary suspect, interaction, and concomitant status. The drug was included in the cases discussed only when it was deemed a “primary suspect”.

### Statistical analysis

To identify potential adverse event signals, we used four disproportionality analysis methods: reporting odds ratio (ROR), proportional reporting ratio (PRR), Bayesian confidence propagation neural network (BCPNN), and Multi-item gamma Poisson Shrinker (MGPS). Each method utilized signal detection parameters derived from 2×2 contingency tables, detailed in Supplementary Table S1. The specific formulas and criteria for signal generation are provided in Supplementary Table S2. We considered a signal valid only if it met the criteria across all four disproportionality analysis methods, thereby indicating a potential association between the drug and proteinuria.

### Logistic regression analysis

All relevant patient information, including gender, age, and weight, was extracted from the FAERS reports, and only reports with complete data were analyzed. Univariate logistic analysis of suspected drugs was conducted utilizing ROR>1, a>100, and a lower limit of the 95% confidence interval for P adjustment <0.01. Drugs with P<0.01 in the one-way analysis were utilized in the least absolute shrinkage and selection operator (LASSO) regression. The LASSO screening for drugs causing adverse events was combined with the patient's basic information as the independent variable, and subsequently, multifactorial logistic regression was used to see if the drug was a risk factor for the adverse reaction. Sex was coded as a binary variable (female as reference). Age was classified into five categories: <18, 18-40, 41-60, 61-80, and >80 years, with the <18 group serving as the reference. Body weight was divided into two categories: 50-100 kg and <50 or >100 kg, using the 50-100 kg group as the reference. Observations with missing values in these variables were excluded from the analysis. We assessed the performance of the best model using the area under the receiver operator characteristic curve (AUC and ROC). Furthermore, we analyzed the duration of drug use prior to the onset of drug-related proteinuria and compared onset times across various medications. All analyses were conducted by R (version 4.4.3) and Microsoft excel 2021.

## Results

### Baseline information on drug-related proteinuria

Data on proteinuria as an adverse event were collected from the first quarter of 2004 to the end of 2024, resulting in a total of 16,355 cases. Table 1 presents the baseline characteristics of drug-related adverse events leading to proteinuria. Among these cases, 7,157 were reported in males, while 6,193 (37.87%) were reported in females. The average age of the patients was 66 years, with an average weight of 86 kg. The highest numbers of reported cases came from the USA, Japan, France, Canada, and Germany, with totals of 5,311 (32.47%), 2,777 (16.98%), 992 (6.07%), 670 (4.10%), and 633 (3.87%) cases, respectively. The findings also revealed a steady increase in adverse event reports over the years, with the highest number documented in the past four years (5,789 cases, 35.40%). Most adverse events occurred within the 18-64 age range, accounting for 6,651 cases (40.67% of the total). Furthermore, a significant proportion of these events were concentrated in patients with a weight range of 51-75 kg, which accounted for 1,269 cases (7.76%).

**Table 1:** Basic information of the patients who experienced drug-related adverse events leading to proteinuria.

Characteristics	Adverse event (n)	Component ratio
Gender		
Male	7157	43.76%
Female	6193	37.87%
Missing	2285	14.00%
Age		
<18	1274	7.79%
18-64	6651	40.67%
65-85	3396	20.76%
>85	179	1.09%
Missing	518	3.17%
Weight (kg)		
<25	65	0.40%
25-50	364	2.23%
51-75	1269	7.76%
>76	755	4.62%
Missing	2367	14.47%
Reporting year		
2004-2008	1523	9.50%
2009-2012	1707	10.65%
2013-2016	2749	17.15%
2017-2020	4594	28.67%
2021-2024	5789	35.40%
Country		
United States	5311	32.47%
Japan	2777	16.98%
France	992	6.07%
Canada	670	4.10%
Germany	633	3.87%
Others	5972	36.51%

A total of 16,635 individual reports presenting with drug-related proteinuria were identified from FDA Adverse Event Reporting System (FAERS).

### Drugs associated with proteinuria

**Figure 1:**
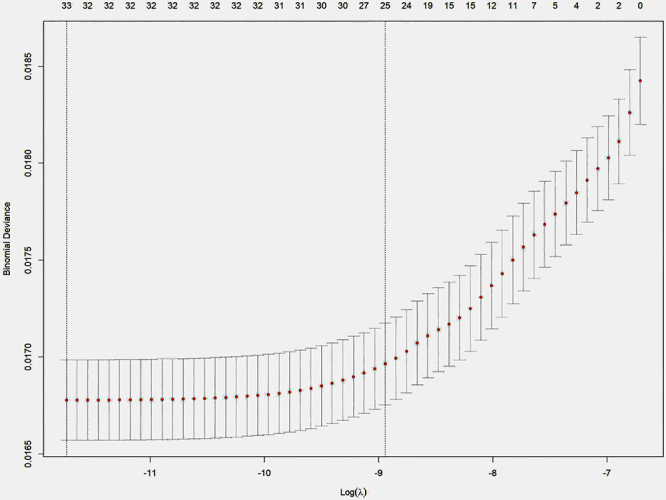
Selection of drugs associated with proteinuria by LASSO (least absolute shrinkage and selection operator) regression. Ten-fold cross-validation curve was used to determine the optimal λ value for model selection. The left dashed line indicates the λ that gives the minimum mean cross-validated error (λ_min), and the right dashed line represents the largest λ within one standard error of the minimum (λ_1se).

**Figure 2:**
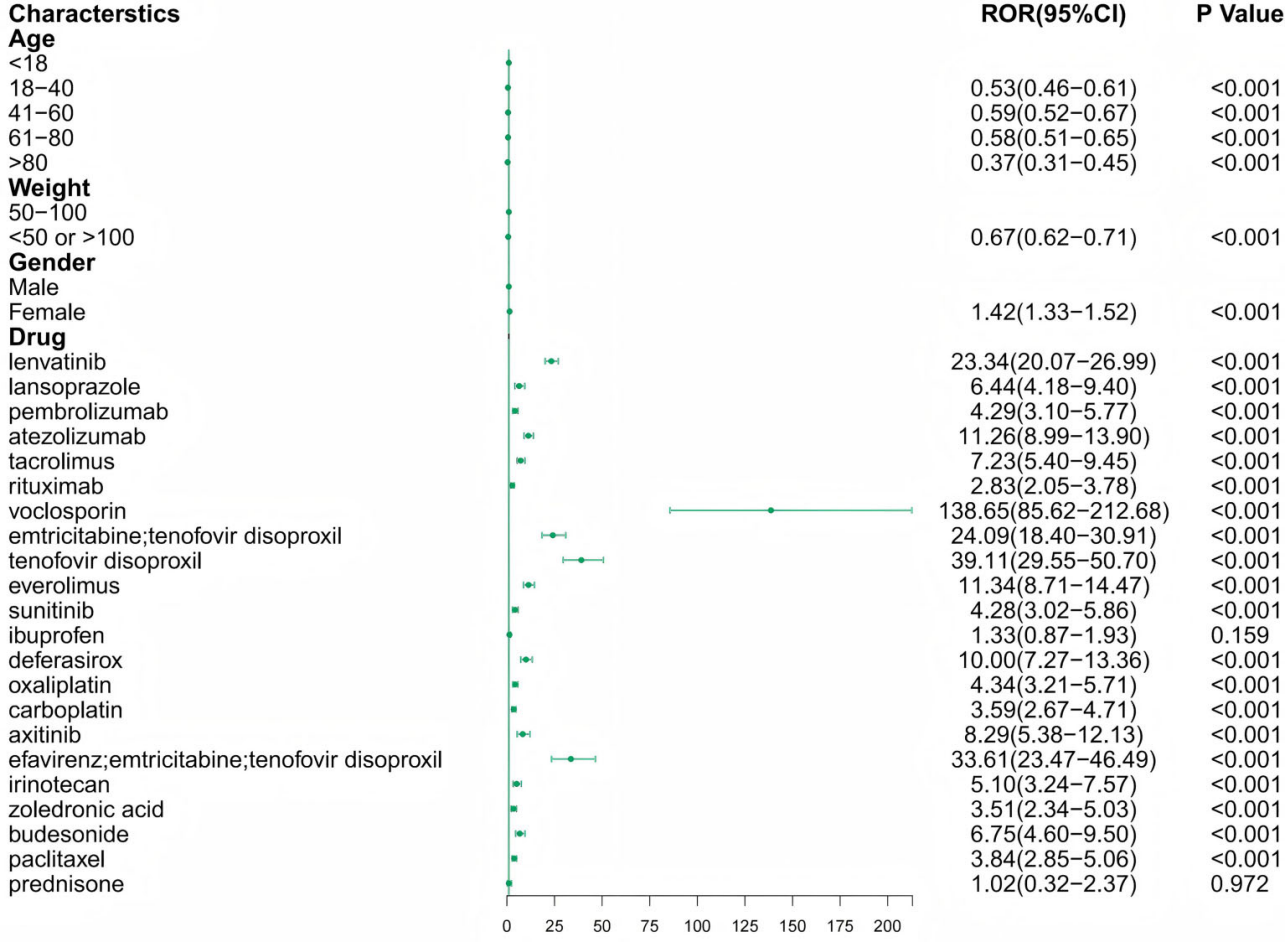
Multivariate logistic regression analysis of drugs associated with proteinuria. Forest plot showing the odds ratios (ORs) and 95% confidence intervals (CIs) for the association between each drug and the occurrence of proteinuria, adjusted for sex, age category, and weight category. Age was classified as <18, 18-40, 41-60, 61-80, and >80 years (<18 as reference), and body weight was classified as 50-100 kg *vs* <50 or >100 kg (50-100 kg as reference).

**Figure 3:**
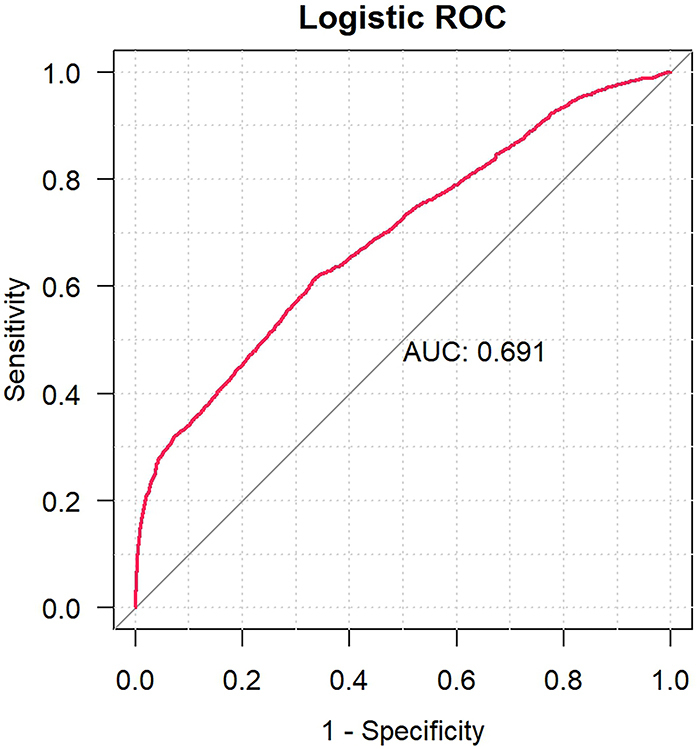
ROC curve of drug-related proteinuria. ROC: receiver operating characteristic; AUC: area under the curve.

A total of 864 drugs were identified as being associated with proteinuria based on the study data. The data were summarized and analyzed using one-way analysis for suspected drugs, with a lower 95% confidence interval of the relative odds ratio exceeding 1 and a P-adjustment of less than 0.01. A final LASSO regression analysis identified a total of 22 drugs from the positive data set (Figure 1). Subsequently, we conducted multivariate logistic analysis and identified 21 drugs (Figure 2). The AUC of the logistic model was 0.691 (Figure 3), showing relatively good predictive accuracy.

Furthermore, we divided 21 drugs into seven types, including anticancer drugs, immunosuppressants, anti-inflammatory drugs, antiviral drugs, orthopedic system drug, digestive system drug, and hematologic system drug (Table 2). The results revealed that certain anticancer agents, immunosuppressants, and antiviral drugs were significantly associated with proteinuria. Voclosporin exhibited the strongest signal, with a ROR of 63.57 (95%CI: 55.92-72.27), indicating a highly probable association. Lenvatinib also showed a strong signal with an ROR of 41.01 (95%CI: 38.05-44.21). Among immune checkpoint inhibitors, atezolizumab and pembrolizumab had RORs of 16.73 and 8.4, respectively, suggesting a moderate association. The antiviral drug tenofovir disoproxil demonstrated a high signal with an ROR of 12.61 (95%CI: 11.07-14.38), consistent with its known nephrotoxic profile. Notably, the gastrointestinal drug lansoprazole also showed a strong signal (ROR=27.22; 95%CI: 25.09-29.52), indicating a potentially underestimated risk.

**Table 2:** Statistical values and distribution of drugs associated with proteinuria.

Drugs	N	ROR (95%CI)	PRR (χ^2^)	EBGM (EBGM05)	IC (IC025)
Anticancer drugs					
Lenvatinib	738	41.01 (38.05-44.21)	39.68 (26593.71)	37.93 (35.63)	5.25 (5.14)
Rituximab	286	3.18 (2.83-3.58)	3.18 (419.48)	3.14 (2.85)	1.65 (1.48)
Voclosporin	250	63.57 (55.92-72.27)	60.31 (14370.77)	59.4 (53.36)	5.89 (5.7)
Everolimus	204	6.32 (5.5-7.26)	6.29 (897.67)	6.23 (5.55)	2.64 (2.44)
Sunitinib	154	4.8 (4.1-5.63)	4.79 (457.48)	4.75 (4.16)	2.25 (2.02)
Oxaliplatin	142	5.16 (4.37-6.08)	5.14 (469.34)	5.1 (4.44)	2.35 (2.11)
Carboplatin	125	3.29 (2.76-3.92)	3.28 (196.99)	3.26 (2.82)	1.71 (1.45)
Axitinib	124	9.54 (7.99-11.4)	9.47 (933.4)	9.41 (8.11)	3.23 (2.97)
Irinotecan	118	10.3 (8.58-12.35)	10.21 (974.55)	10.15 (8.71)	3.34 (3.08)
Paclitaxel	103	3.29 (2.71-4)	3.29 (163)	3.27 (2.78)	1.71 (1.43)
Immunosuppressants					
Pembrolizumab	346	8.4 (7.55-9.34)	8.34 (2190.8)	8.19 (7.49)	3.03 (2.88)
Atezolizumab	312	16.73 (14.95-18.73)	16.51 (4463.06)	16.21 (14.75)	4.02 (3.85)
Tacrolimus	310	6.04 (5.39-6.75)	6.01 (1270.92)	5.91 (5.38)	2.56 (2.4)
Budesonide	110	6.0 (4.97-7.24)	5.97 (452.45)	5.94 (5.07)	2.57 (2.29)
Anti-inflammatory drugs					
Ibuprofen	153	2.21 (1.88-2.59)	2.21 (100.05)	2.19 (1.92)	1.13 (0.9)
Prednisone	101	3.28 (2.7-3.99)	3.27 (158.63)	3.26 (2.77)	1.7 (1.42)
Antiviral drugs					
Tenofovir disoproxil	230	12.61 (11.07-14.38)	12.49 (2398.23)	12.32 (11.05)	3.62 (3.43)
Efavirenz/Emtricitabine/Tenofovir disoproxil	124	14.74 (12.34-17.61)	14.57 (1556.32)	14.46 (12.46)	3.85 (3.59)
Orthopedic system drug					
Zoledronic acid	116	2.23 (1.86-2.68)	2.23 (78.33)	2.22 (1.91)	1.15 (0.88)
Digestive system drug					
Lansoprazole	619	27.22 (25.09-29.52)	26.62 (14700.84)	25.65 (23.97)	4.68 (4.56)
Hematologic system drug					
Deferasirox	146	7.54 (6.4-8.88)	7.5 (815.66)	7.44 (6.49)	2.9 (2.66)

ROR: reporting odds ratio; PRR: proportional reporting ratio; EBGM: empirical Bayes geometric mean; EBGM05: lower limit of 95%CI of EBGM; IC: information component; IC025: lower limit of 95%CI of the IC.

After adjustment for sex, age, and body weight, several demographic covariates showed significant associations with the occurrence of drug-related proteinuria. Compared with individuals aged <18 years, those aged 18-40 years (P<0.01), 41-60 years (P<0.01), 61-80 years (P<0.01), and >80 years (P<0.01) were less likely to develop proteinuria. Patients with body weight outside the 50-100 kg range also exhibited a lower risk (P<0.01) compared with those within the reference range. In contrast, female patients showed a significantly higher likelihood of proteinuria than males (P<0.01). These findings suggest that younger age, male sex, and moderate body weight are associated with a higher probability of reporting drug-induced proteinuria (Figure 2).

### Time between the use of drugs and the occurrence of adverse reactions

**Figure 4:**
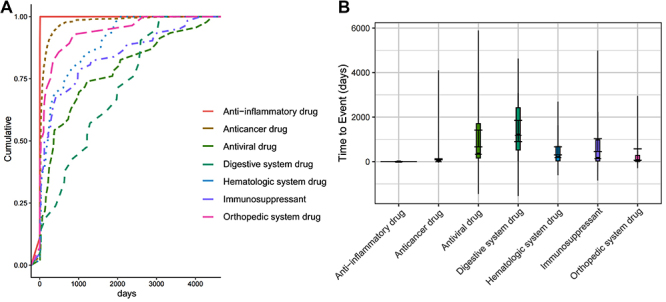
Adverse event onset time distribution of drug-related proteinuria across different drug systems. **A**, Cumulative incidence curves showing the temporal risk of proteinuria induced by different drug systems. **B**, Violin plots illustrating the distribution and variation in onset times of proteinuria among different drug categories. Drug classes included antiviral drugs, anticancer drugs, digestive system drugs, orthopedic system drugs, hematologic system drugs, anti-inflammatory drugs, and immunosuppressants.

We further analyzed the time to occurrence (TTO) of proteinuria across different drug categories using cumulative incidence curves, boxplots, and descriptive statistics. The results revealed substantial variation in the onset timing of proteinuria depending on drug class (Table 3 and Figure 4).

**Table 3:** Adverse event onset time distribution of drug-related proteinuria in different systems.

System	Mean	Q1	Q3
Antiviral drug	1040.4	163.0	1706.0
Anticancer drug	106.2	12.0	83.0
Digestive system drug	1376.8	523.0	2423.0
Orthopedic system drug	319.2	6.8	280.8
Hematologic system drug	483.5	34.0	680.0
Anti-inflammatory drug	5.4	5.0	5.0
Immunosuppressant	744.6	27.3	977.0

Mean: Arithmetic mean; Q1: first quartile; Q3: third quartile.

Anti-inflammatory drugs exhibited the earliest onset, with a mean TTO of 5.4 days and a very narrow interquartile range (Q1-Q3: 5.0-5.0), indicating rapid and consistent occurrence. Similarly, anticancer drugs showed a relatively early onset (mean=106.2 days, Q1-Q3: 12.0-83.0), although with greater variability, as illustrated by the wider box in the boxplot and the sharp early rise in the cumulative curve. In contrast, digestive system drugs and antiviral drugs were associated with much longer onset times, with mean TTOs of 1376.8 and 1040.4 days, respectively. Their interquartile ranges (digestive: Q1-Q3: 523.0-2423.0; antiviral: Q1-Q3: 163.0-1706.0) suggest a wide temporal distribution of adverse events. These patterns were consistent with their flatter cumulative incidence curves and broader spread in boxplots. Immunosuppressants and hematologic system drugs presented intermediate profiles, with mean TTOs of 744.6 and 483.5 days, respectively. Orthopedic system drugs showed moderate onset (mean=319.2 days), but with early Q1 [6.8] suggesting some early-onset cases.

These findings underscore that the timing of proteinuria onset varies substantially across drug classes. Early-onset events with anti-inflammatory and anticancer drugs highlight the need for prompt monitoring soon after initiation, whereas delayed-onset patterns in digestive and antiviral drugs suggest the importance of long-term renal surveillance during treatment.

## Discussion

This study systematically evaluated the risk of drug-induced proteinuria using data from the FAERS database, identifying multiple high-risk medications, especially within the domains of oncology and immunotherapy. Among the strongest signals were anti-angiogenic agents and immune checkpoint inhibitors (ICIs), both of which are widely used in cancer treatment. These findings align with current clinical observations and underscore the increasing relevance of nephrotoxicity as a limiting factor in long-term therapeutic strategies. The variability in onset time further emphasizes the complexity of proteinuria pathogenesis and the necessity for tailored monitoring strategies across different drug classes.

Anti-angiogenic agents in anticancer drugs such as lenvatinib, axitinib, and sunitinib demonstrated strong signal associations with proteinuria, indicating that this drug class may pose a substantial risk for its development. Lenvatinib, a multi-targeted tyrosine kinase inhibitor, frequently causes proteinuria as a common adverse event (14). A two-year study reported a general decline in estimated glomerular filtration rate (eGFR) among patients receiving Lenvatinib, which improved following drug discontinuation - suggesting that Lenvatinib-induced renal impairment may be at least partially reversible (15). This observation supports the notion that proteinuria may represent one phenotypic manifestation of VEGF inhibitor-related nephrotoxicity. Accordingly, irrespective of proteinuria severity, continuous monitoring of renal function is essential for optimal clinical management.

VEGF inhibitors such as bevacizumab have been shown to induce proteinuria in a dose-dependent manner, typically without significant deterioration in renal function (16). However, compared with bevacizumab, Lenvatinib - due to its stronger VEGF-inhibitory activity - appears to carry a higher risk of proteinuria. Other VEGF/VEGFR inhibitors, including axitinib and sunitinib, are similarly associated with proteinuria. These agents disrupt VEGF-mediated signaling between glomerular endothelial cells and podocytes, which is critical for maintaining the integrity of the glomerular filtration barrier (17). This disruption may result in microvascular injury, endothelial dysfunction, and increased glomerular permeability, potentially progressing to pathological changes such as glomerulosclerosis or focal segmental glomerulosclerosis (FSGS) with prolonged exposure (18,19). In clinical practice, patients receiving VEGF pathway inhibitors should undergo baseline assessment of renal function, followed by regular monitoring of proteinuria and eGFR during treatment. In cases of moderate to severe proteinuria, dose adjustment or temporary discontinuation should be considered. For high-risk individuals, including those with pre-existing chronic kidney disease, hypertension, or diabetes, more intensive and individualized nephroprotective strategies are warranted.

In addition to anti-angiogenic agents, this study identified significant signal associations between ICIs, such as atezolizumab and pembrolizumab, and proteinuria. ICIs enhance antitumor immunity by blocking inhibitory pathways - including PD-1/PD-L1 and CTLA-4 - but this immune activation may also lead to immune-related adverse events (irAEs), commonly affecting the skin, gastrointestinal tract, and endocrine system. Renal irAEs, though less frequent, are increasingly reported (20). Acute interstitial nephritis is the most commonly documented ICI-associated renal lesion (21). However, glomerular pathologies - such as minimal change disease, membranous nephropathy, FSGS, and immune complex glomerulonephritis - have also been observed (22). These conditions typically present with proteinuria or nephrotic syndrome, often emerging within weeks to months of ICI initiation (23), consistent with the median onset time observed in this study. The pathogenesis of ICI-induced renal injury remains incompletely understood. Proposed mechanisms include the development of autoantibodies, cytotoxic T cell-mediated tissue damage, and the unmasking of latent autoimmune conditions (24). The detection of anti-neutrophil cytoplasmic antibodies (ANCA) and anti-PLA2R antibodies in one case report further supports an immune-mediated etiology (25). Clinically, early evaluation is essential when ICI-associated proteinuria or renal injury is suspected. Recommended assessments include urinalysis, renal function tests, and renal biopsy to determine the pathological subtype. Management typically involves temporary discontinuation of ICIs and corticosteroid therapy. In severe or refractory cases, additional immunosuppressants such as mycophenolate mofetil or cyclophosphamide may be necessary. Reinitiation of ICIs should be approached cautiously, balancing oncologic benefit against the risk of recurrent nephrotoxicity. While ICIs have markedly improved outcomes across various malignancies, their potential for renal toxicity warrants careful consideration. Comprehensive pre-treatment evaluation of autoimmune history and concomitant nephrotoxic medications (e.g., PPIs, NSAIDs) (26,27) are also essential to optimize therapeutic outcomes and minimize adverse effects.

Everolimus, an mTOR pathway inhibitor, is widely employed in the treatment of various solid tumors and hematologic malignancies. In this study, everolimus exhibited a significant association with proteinuria, consistent with its known nephrotoxicity in clinical settings (28). The mTOR signaling pathway is essential for maintaining podocyte integrity and function; its inhibition may induce podocyte apoptosis, reduce nephrin expression, and compromise the glomerular filtration barrier, ultimately leading to proteinuria. Notably, everolimus-induced proteinuria often demonstrates a dose-dependent pattern (29). Co-administration of angiotensin converting enzyme inhibitors (ACEIs) or angiotensin receptor blockers (ARBs) has been reported to mitigate renal toxicity in some patients (30,31). Accordingly, regular monitoring of proteinuria and renal function is recommended during mTOR inhibitor therapy, along with a thorough evaluation of potential drug-drug interactions that may exacerbate nephrotoxicity.

Cytotoxic chemotherapeutic agents, including carboplatin, oxaliplatin, paclitaxel, and irinotecan, also demonstrated moderate signal associations with proteinuria in this analysis. Unlike targeted therapies, their nephrotoxic effects are typically indirect, involving oxidative stress, tubular injury, inflammation, or nephrotoxic metabolites, which may secondarily disrupt the glomerular filtration barrier (32). Additionally, factors such as combination regimens, cumulative drug exposure, and underlying comorbidities can significantly influence proteinuria risk. Therefore, in patients receiving cytotoxic chemotherapy - particularly older adults or those concurrently exposed to other nephrotoxic agents - more frequent urinalysis and eGFR monitoring is warranted to enable early detection and prevention of renal injury.

In addition to antineoplastic agents, this study identified several non-oncologic drugs significantly associated with proteinuria, underscoring the importance of renal safety considerations beyond oncology. Tenofovir disoproxil and its fixed-dose combination formulations (e.g., efavirenz/emtricitabine/tenofovir disoproxil), widely used for HIV and HBV treatment, demonstrated strong signal associations with proteinuria, consistent with their established nephrotoxicity (33). Tenofovir induces proximal tubular injury via mitochondrial toxicity, leading to clinical manifestations such as hypophosphatemia, aminoaciduria, glycosuria, and proteinuria, with severe cases progressing to Fanconi syndrome (34,35). Routine monitoring of urinalysis, serum creatinine, and phosphate levels is recommended during long-term therapy. In the event of renal impairment, dose adjustment or transition to the less nephrotoxic tenofovir alafenamide should be considered (36).

Zoledronic acid, a potent bisphosphonate used for osteoporosis and malignancy-associated bone disease, was also associated with proteinuria (37). While its primary nephrotoxic effect involves acute tubular necrosis, emerging evidence suggests potential glomerular involvement, particularly with rapid infusion or concomitant nephrotoxic agents (38). One case report describes a patient who developed extensive renal tubular dysfunction ultimately diagnosed as Fanconi syndrome and distal renal tubular acidosis secondary to zoledronic acid therapy, following five years of 4-week cyclic zoledronic acid infusion treatment (39). Notably, proteinuria served as a precursor manifestation of Fanconi syndrome, highlighting the need for vigilant monitoring in such clinical scenarios. Deferasirox, an oral iron chelator used for transfusion-related iron overload, also demonstrated a moderate association with proteinuria (40). Its nephrotoxic effects may involve tubular toxicity, oxidative stress, and impairment of glomerular filtration. Reversible proteinuria and eGFR decline have been reported during therapy. Therefore, close renal monitoring is essential, and treatment adjustments or temporary discontinuation may be warranted in the event of renal dysfunction.

This study is limited by the potential influence of single or several concomitant drugs that may skew the results in cases with proteinuria. Consequently, it is challenging to ascertain whether proteinuria is attributable to medication or individual factors. Elaborating on this aspect is challenging due to the frequent occurrence of numerous patients utilizing various medications simultaneously at varied intervals, making it impossible to ascertain the specific indication linked to each prescription. Consequently, examining solely one medicine in our study does not provide a comprehensive representation of the data on immunosuppressants referenced in our article.

## Conclusion

In conclusion, our pharmacovigilance analysis of FAERS data over two decades revealed a distinct pattern of drug-induced proteinuria risk, primarily driven by targeted anticancer therapies, immunosuppressants, and antiviral agents. The identification of strong signal associations and time-to-onset characteristics provides critical insights into early detection and management strategies. Clinicians should remain vigilant during drug initiation and long-term use, particularly for agents such as lenvatinib, voclosporin, and tenofovir disoproxil. Enhanced renal function monitoring, dose adjustments, and personalized risk assessments are essential to minimize nephrotoxicity and ensure medication safety in vulnerable populations.

## Data Availability

The datasets presented in this study can be found in online repositories. The names of the repository/repositories and accession number(s) can be found at FAERS Publish Dashboard (https://www.fda.gov/drugs/questions-and-answers-fdas-adverse-event-reporting-system-faers/fda-adverse-event-reporting-system-faers-public-dashboard).

## Supplementary Materials

Supplementary Material Click here to view [pdf].
