# Supplementary material for: Drug-related proteinuria: a vigilance analysis based on the FAERS database
Source: Braz J Med Biol Res. 2026 Mar 2;59:e15097. doi: 10.1590/1414-431X2025e15097 (PMC12990312; doi:10.1590/1414-431X2025e15097)
Supplement: Supplementary Material [file 1414-431X-bjmbr-59-e15097-suppl.pdf]

Table S1. Two by two table for signal detection.

Type of drug	Target adverse reaction reports	Other adverse reaction reports	Sum
Target drug	a	b	a+b
Other drugs	c	d	c+d
Sum	a+c	b+d	N=a+b+c+d

a: number of reports containing both the target drug and target adverse reaction reports; b: number of reports containing other adverse reaction reports of the target drug; c: number of reports containing the target adverse reaction reports of other drugs; d: number of reports containing other drugs and other adverse reaction reports; N: number of reports.

Table S2. Four major algorithms were used for signal detection.

Algorithms	Equation	Criteria
ROR	ROR=(a/c)/(b/d)<br>$95\%CI=e^{\ln(ROR) \pm 1.96(1/a+1/b+1/c+1/d)^{\wedge}0.5}$	95%CI>1, N≥3
PRR	PRR=[a/(a + b)]/[c/(c + d)]<br>χ^2=[(ad-bc)^2](a + b + c + d)/[(a + b)(c + d)(a + c)(b + d)]	PRR≥2, χ^2≥4, N≥3
MGPS	EBGM=a(a + b + c + d)/[(a + c)(a + b)]<br>$EBGM05=e^{\ln(EBGM)-1.64(1/a+1/b + 1/c + 1/d)^{\wedge}0.5}$	EBGM05>2
BCPNN	$IC=\log_2 a(a+b+c+d)/[(a+c)(a+b)]$<br>$IC025=e^{\ln(IC)-1.96(1/a + 1/b + 1/c + 1/d)^{\wedge}0.5}$	IC025>0

ROR: reporting odds ratio; PRR: proportional reporting ratio; MGPS, multi-item gamma Poisson shrinker; BCPNN: Bayesian confidence propagation neural network. a: number of reports containing both the target drug and target adverse reaction reports; b: number of reports containing other adverse reaction reports of the target drug; c: number of reports containing the target adverse reaction reports of other drugs; d: number of reports containing other drugs and other adverse reaction reports. 95%CI: 95% confidence interval; N: number of reports; χ^2: chi-squared; EBGM: empirical Bayesian geometric mean; EBGM05: lower limit of 95%CI of EBGM; IC: information component; IC025: lower limit of 95%CI of the IC.
